# Prognostic value of resected lymph nodes numbers for Siewert II gastroesophageal junction cancer

**DOI:** 10.18632/oncotarget.23540

**Published:** 2017-12-20

**Authors:** Sanchuan Lai, Tingting Su, Xingkang He, Zhenghua Lin, Shujie Chen

**Affiliations:** ^1^ Sir Run Run Shaw Hospital, Zhejiang University School of Medicine, Department of Gastroenterology, Hangzhou, Zhejiang 310016, China; ^2^ Institute of Gastroenterology, Zhejiang University, Hangzhou, Zhejiang 310016, China

**Keywords:** resected lymph nodes, Siewert type II, gastroesophageal junction cancer, lymph node ratio

## Abstract

We aim to evaluate whether resected lymph nodes (RLNs) numbers have prognostic value in patients with gastroesophageal junction cancers (GEJ, Siewert type II). Patients with gastroesophageal junction cancers were identified from the Surveillance Epidemiology and End Results (SEER) registry between 1988 to 2013. Multivariate Cox regression analyses and Kaplan–Meier method were performed to analyze risk factors for overall survival (OS) and cause-specific survival(CSS). A total of 8396 patients who underwent surgeries and had reginal lymph nodes examined were identified. Kaplan–Meier analysis indicated that more numbers of resected lymph nodes (RLNs) were associated with better survival. The five-year OS rates for 1–20 and 21–90 RLNs were 26.8% and 32.4%, with a median survival time of 62 and 72 months, respectively (*P* < 0.001). The five-year CSS rates were 32.2% and 37.2% in each group, with median survival time of 90 and 101 months, respectively (*P* < 0.001). Cox regression multivariate analysis showed that year of diagnosis, age, sex, marital status, grade, seer histology, tumor histology, lymph node ratio (LNR) and RLNs as a categorical variable were all significant prognostic factors for both OS and CSS. RLN count is an independent prognostic factor for Siewert type II GEJ cancer patients and patients can achieve better overall and cancer-specific survival with more than 20 RLNs dissected.

## INTRODUCTION

Gastroesophageal junction (GEJ) malignancies are among the most common cancer-caused mortality worldwide. Approximately 1.4 million new GEJ cancers are diagnosed every year globally [1]. Its location ranges from the distal esophagus to the proximal stomach. According to the Siewert classification [2]. the GEJ cancers could be divided into 3 subtypes: type I GEJ cancers are 1cm to 5cm above the GEJ, while type II and type III cancers are 1cm to 2cm and 2cm to 5cm below the GEJ, respectively [3]. The 5-year survival for GEJ cancers were 30% with only surgery, which might be attributed to the high recurrence rate of this malignancy and its metastatic potential [4]. As the treatment of gastric cancers is different from that of esophageal cancer, the specific treatment for GEJ cancers remains controversial.

Based on the similarity between Siewert I GEJ cancer and esophageal cancer, Siewert III GEJ cancer and gastric cancer, the treatment for Siewert I and III type cancers mirror those for esophageal and gastric cancer [5–7]. However, the optimal treatment for Siewert type II cancers still remains to be determined [8]. Some authors preferred transhiatal extended gastrectomy for Siewert type II GEJ cancers while others insisted thoracoabdominal esophagectomy should be recommended [5, 9–14]. Yuasa reported that Siewert type II cancers was associated with a significant shorter 5-year survival rates compared with type III (67% with 87%), he also pointed out a higher metastatic potential of Siewert type II cancers than type III [15]. Meanwhile, Siewert type II cancers were reported to have a two-fold lower 5-year survival rate than type III [9]. Accordingly, finding a refined surgical procedure with lymph nodes dissection solely on Siewert type II cancers is imperatively needed.

Lymph nodes resection was considered effective in improving overall survival in several tumors [16–18]. Given the metastatic potential of Siewert type II cancer, completed lymphadenectomy might have therapeutic value considering the removal of positive lymph nodes. As Resected Lymph Nodes (RLN) count is the main evaluation for lymphadenectomy, it might be a prognostic index in Siewert type II cancer.

In this study, we aim to demonstrate whether resected lymph nodes numbers have prognostic value in Siewert type II cancer patients and evaluate the potential effect of lymph node status and Lymph Node Ratio (LNR) on the overall and cancer-specific survival by using The Surveillance, Epidemiology, and Results (SEER) database. The Seer database allows us to examine the survival of patients across the facilities around the United States based on individual characteristics, including sex, age, time of diagnosis, histological subtypes, grade, radiation sequence, number of lymph nodes examined/removed, number of positive lymph nodes, marital status, *et al*.

## RESULTS

### Patient characteristics and lymph node resection

A total of 8396 patients who underwent surgeries and had reginal lymph nodes examined were identified from 1988 to 2013. Clinical characteristics of patients were shown in Table 1. With a median age of 54 years old (range from 18 to 95), 89.2% patients were white people, 81.8% were male and 70.0% were married. The main tumor histology presented in this study was adenocarcinoma (81.6%), with 12.8% of cystic and mucinous neoplasms and only 2.0% of squamous cell neoplasms. As to the SEER histology stage, 51.0% were reginal, 33.5% were localized and the rest 15.0% were distant. Most of Siewert type II GEJ cancer were poorly differentiated (52.2%), with only 6.0% well differentiated and 33.2% moderately differentiated. The mean LNR in all patients was 0.319, and 3145 (37.6%) patients were node –negative, while the other 5251 (62.4%) had lymph node metastasis.

**Table 1:** Baseline characteristics of patients with Siewert type II GEJ cancer

Characteristic	*n*	1–10 RLNs (%)	11–20 RLNs (%)	21–30 RLNs (%)	31–90 RLNs (%)	*P*-value
Year of diagnosis						
1988–1992	886	501 (14.4)	288 (9.3)	75 (6.4)	22 (3.4)	*P* < 0.05
1993–1997	1077	542 (15.6)	374 (12.1)	117 (10.0)	44 (6.8)	
1998–2002	1755	889 (25.6)	577 (18.6)	180 (15.3)	109 (16.8)	
2003–2007	2153	802 (23.1)	813 (26.2)	338 (28.8)	200 (30.8)	
2008–2013	2525	735 (21.2)	1050 (33.8)	465 (39.6)	275 (42.3)	
Race						
Black	344	136 (3.9)	120 (3.9)	48 (4.1)	40 (6.2)	*P* < 0.05
White	7382	3095 (89.2)	2748 (88.6)	1000 (85.1)	539 (82.9)	
Other	651	233 (6.7)	225 (7.3)	124 (10.6)	69 (10.6)	
Age						
≤60	3159	1240 (35.7)	1172 (37.8)	474 (40.3)	273 (42.0)	*P* = 0.003
>60	5237	2229 (64.3)	1930 (62.2)	701 (59.7)	377 (58.0)	
Sex						
Male	6781	2837 (81.8)	2504 (80.7)	914 (77.8)	526 (80.9)	*P* = 0.029
Female	1615	632 (18.2)	598 (19.3)	261 (22.2)	124 (19.1)	
Tumor histology						
Adenocarcinoma	6845	2832 (81.6)	2553 (82.3)	951 (80.9)	509 (78.3)	*P* < 0.05
Cystic and mucinous	1205	445 (12.8)	455 (14.7)	181 (15.4)	124 (19.1)	
Squamous	132	71 (2.0)	39 (1.3)	16 (1.4)	6 (0.9)	
Other	214	121 (3.5)	55 (1.8)	27 (2.3)	11 (1.7)	
Grade						
Well differentiated	417	209 (6.0)	127 (4.1)	55 (4.7)	26 (4.0)	*P* < 0.05
Moderately differentiated	2717	1153 (33.2)	994 (32.0)	373 (31.7)	197 (30.3)	
Poorly differentiated	4600	1811 (52.2)	1752 (56.5)	648 (55.1)	389 (59.8)	
Undifferentiated	211	74 (2.1)	76 (2.5)	43 (3.7)	18 (2.8)	
Seer histology						
Reginal	4836	1769 (51.0)	1901 (61.3)	749 (63.7)	417 (64.2)	*P* < 0.05
Localized	2263	1163 (33.5)	736 (23.7)	242 (20.6)	122 (18.8)	
Distant	1264	521 (15.0)	455 (14.7)	179 (15.2)	109 (16.8)	
Unstaged	33	16 (0.5)	10 (0.3)	5 (0.4)	2 (0.3)	
Marital status						
Married	5869	2430 (70.0)	2175 (70.1)	804 (68.4)	460 (70.8)	*P* = 0.860
Not married	2328	959 (27.6)	852 (27.5)	339 (28.9)	178 (27.4)	
Radiation						
Radiation	3369	1351 (38.9)	1314 (42.4)	456 (38.8)	248 (38.2)	*P* = 0.055
No radiation	4906	2071 (59.7)	1744 (56.2)	697 (59.3)	394 (60.6)	

RLNs, resected lymph nodes.

All patients were divided into four groups as categorical variables based on their RLNs counts (Group A [1–10], Group B [11–20], Group C [21–30] and Group D [31–90]).

In this retrospective study, RLNs counts were associated with the year of diagnosis (*P* < 0.05), race (*P* < 0.05), age (*P* = 0.003), sex (*P* = 0.029), tumor histology (*P* < 0.05), grade (*P* < 0.05) and SEER histology stage (*P* < 0.05), but was not associated with marital status (*P* = 0.860) and radiation (*P* = 0.055).

### Prognostic analysis

**Figure 1:**
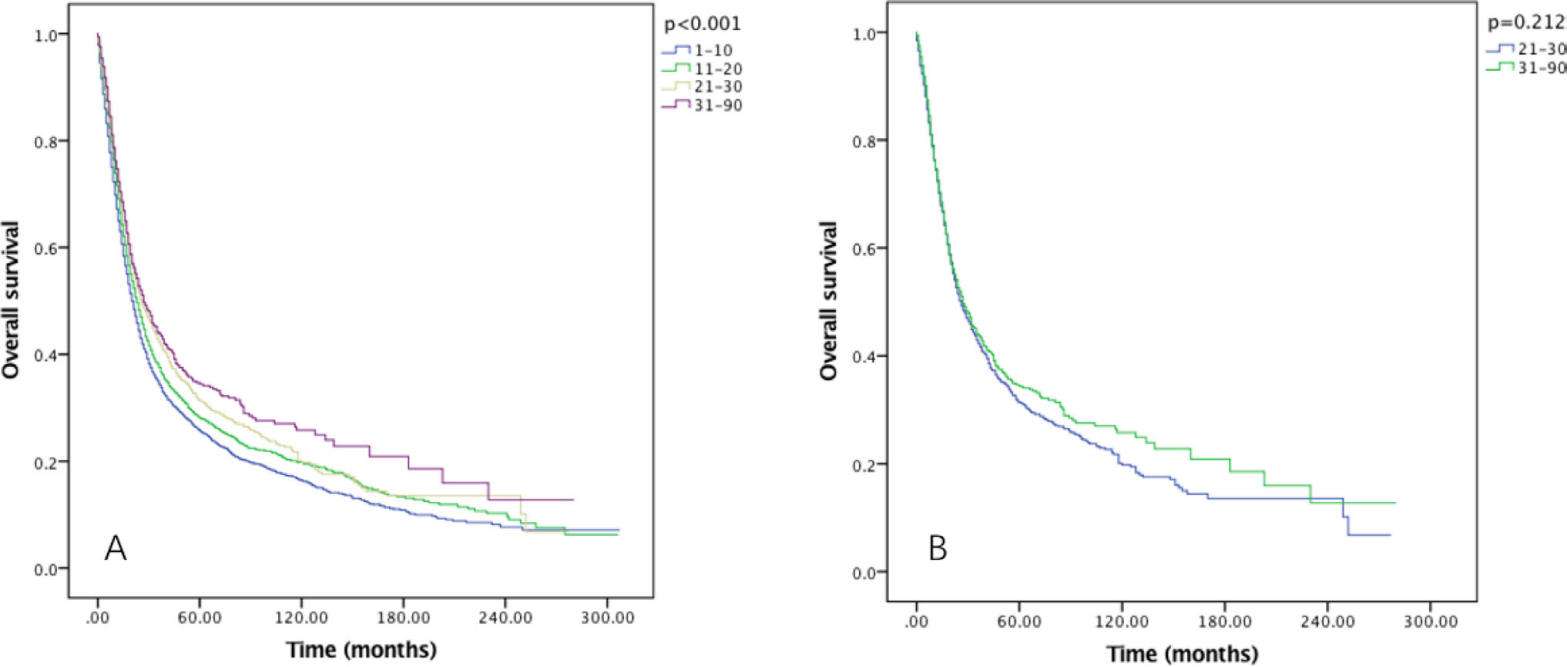
Overall survival of Siewert type II GEJ cancer patients in different RLNs group Kaplan-Meier curves illustrate the survival of patients in four different RLNs group (**A**) and the survival of patients between 21–30 RLNs group and 31–90 RLNs group (**B**) Significance was determined by log-rank analysis.

Though RLNs as a categorical variable was a significant prognostic factor in both OS and CSS (*P* < 0.001), no significance was found when comparing the OS and CSS in Group D (31–90) and Group C (21–30) patients (both *p* > 0.05) using Kaplan–Meier analysis (Figure 1). Multivariate Cox regression analysis also revealed the survival months in Group D was not significantly better than Group C (OS: HR = 0.959, *P* = 0.5; CSS: HR = 0.978, *P* = 0.748). The AJCC staging system suggested at least 15 lymph nodes examined for adequate staging in gastric cancer [19]. Multivariate Cox regression indicated the OS and CSS in 21–90 LNs Group yielded significant better survival than 15–20 LNs Group (OS: HR = 0.904, *P* = 0.019; CSS: HR = 0.891, *P* = 0.013). Therefore, we combined Group A and Group B together as Group 1, Group C and Group D together as Group 2 for further analysis.

Multivariate Cox regression analysis showed that year of diagnosis, age, sex, marital status, grade, seer histology, tumor histology, radiation, LNR and RLNs as a categorical variable were all significant prognostic factors for both OS and CSS (Table 2).

**Table 2:** Multivariate analysis of prognostic factors associated with the survival

Characteristic	OS	95% CI	*P* value	CSS	95% CI	*P* value
	HR			HR		
Year of diagnosis (continuous variable)	0.967	0.963–0.970	*P* < 0.001	0.965	0.961–0.969	*P* < 0.001
Age (continuous variable)	1.022	1.019–1.024	*P* < 0.001	1.015	1.012–1.018	*P* < 0.001
Sex						
Male	1			1		
female	0.858	0.803–0.917	*P* < 0.001	0.897	0.835–0.965	*P* = 0.003
Race						
Black	1			1		
White	0.937	0.824–1.065	*P* = 0.32	1.017	0.880–1.174	*P* = 0.822
Other	0.820	0.701–0.960	*P* = 0.013	0.859	0.721–1.025	*P* = 0.092
Marital status						
Married	1			1		
Not married	1.168	1.111–1.227	*P* < 0.001	1.122	1.062–1.185	*P* < 0.001
Grade						
Well differentiated	1			1		
Moderately differentiated	1.194	1.039–1.372	*P* = 0.013	1.278	1.082–1.509	*P* = 0.004
Poorly differentiated	1.508	1.316–1.729	*P* < 0.001	1.700	1.444–2.002	*P* < 0.001
Undifferentiated	1.462	1.195–1.788	*P* < 0.001	1.660	1.322–2.084	*P* < 0.001
Seer histology						
Regional	1			1		
Localized	0.449	0.419–0.481	*P* < 0.001	0.352	0.324–0.382	*P* < 0.001
Distant	1.563	1.458–1.675	*P* < 0.001	1.618	1.505–1.740	*P* < 0.001
Unstaged	0.481	0.278–0.830	*P* = 0.009	0.464	0.256–0.841	*P* = 0.011
Tumor histology						
Adenocarcinoma	1			1		
Cystic and mucinous neoplasm	1.132	1.053–1.216	*P* = 0.001	1.141	1.057–1.233	*P* = 0.001
Squamous cell neoplasms	1.343	1.105–1.632	*P* = 0.003	1.339	1.076–1.665	*P* = 0.009
Other	1.046	0.891–1.228	*P* = 0.581	1.069	0.898–1.273	*P* = 0.453
Radiation						
Radiation	1			1		
No radiation	1.173	1.112–1.236	*P* < 0.001	1.169	1.104–1.238	*P* < 0.001
LNR (continuous variable)	1.021	1.011–1.031	*P* < 0.001	1.021	1.011–1.031	*P* < 0.001
RLNs						
1–20	1			1		
21–90	0.836	0.783–0.893	*P* < 0.001	0.823	0.766–0.884	*P* < 0.001

OS, overall survival; CSS, cause-specific survival; HR, hazard ratio; CI, confidence interval; LNR, lymph node ratio; RLNs, resected lymph nodes.

After comparing the different lymph node ratio in Group 1 and Group 2, significant higher LNR was observed in 21–90 RLNs Group (*P* = 0.002)

### Survival in RLNs groups

**Figure 2:**
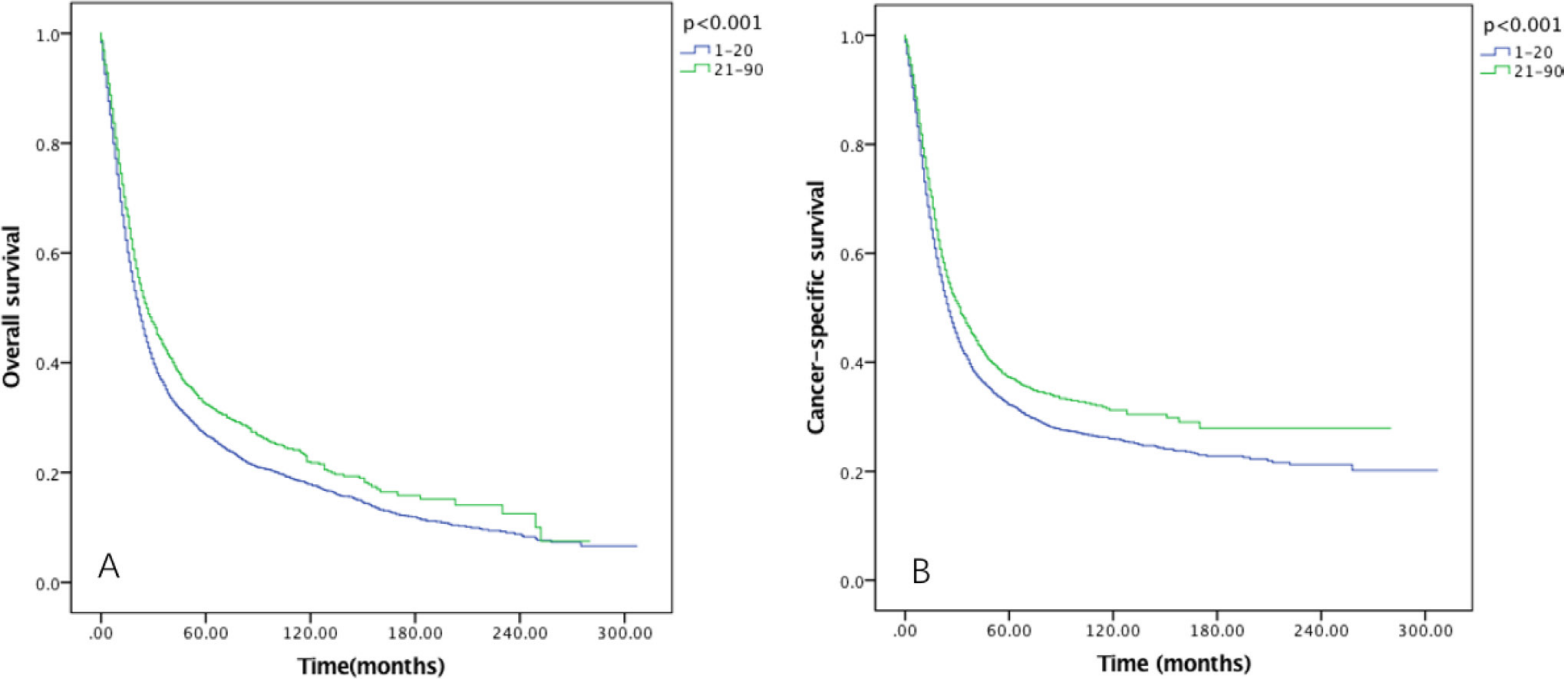
Overall survival (**A**) and cause-specific survival (**B**) of Siewert type II GEJ cancer patients in different RLNs groups. Kaplan-Meier curves illustrate the overall survival (A) and cause-specific survival (B) of patients between 1–20 RLNs group and 21–90 RLNs group. Significance was determined by log-rank analysis.

The five-year OS rates for 1–20 and 21–90 RLNs were 26.8% and 32.4%, with a median survival time of 62 and 72 months, respectively (*P* < 0.001) (Figure 2A). While the five-year CSS rates were 32.2% and 37.2% in each group, with median survival time of 90 and 101 months, respectively (*P* < 0.001) (Figure 2B).

**Figure 3:**
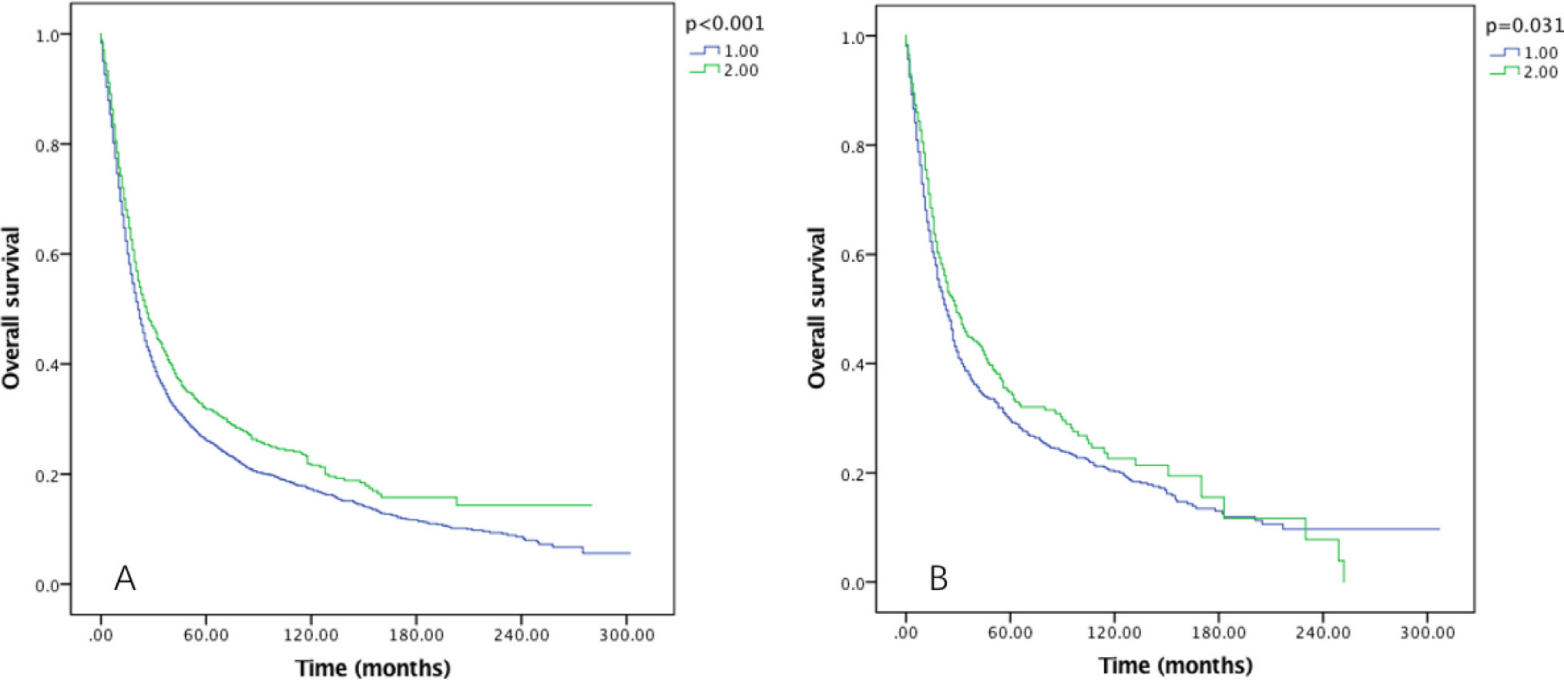
Overall survival of Siewert type II GEJ cancer patients in different RLNs groups stratified by sex (**A**) male, (**B**) female. Kaplan-Meier curves illustrate the overall survival of patients between 1–20 RLNs group and 21–90 RLNs group in male (A) and female (B). Significance was determined by log-rank analysis.

**Figure 4:**
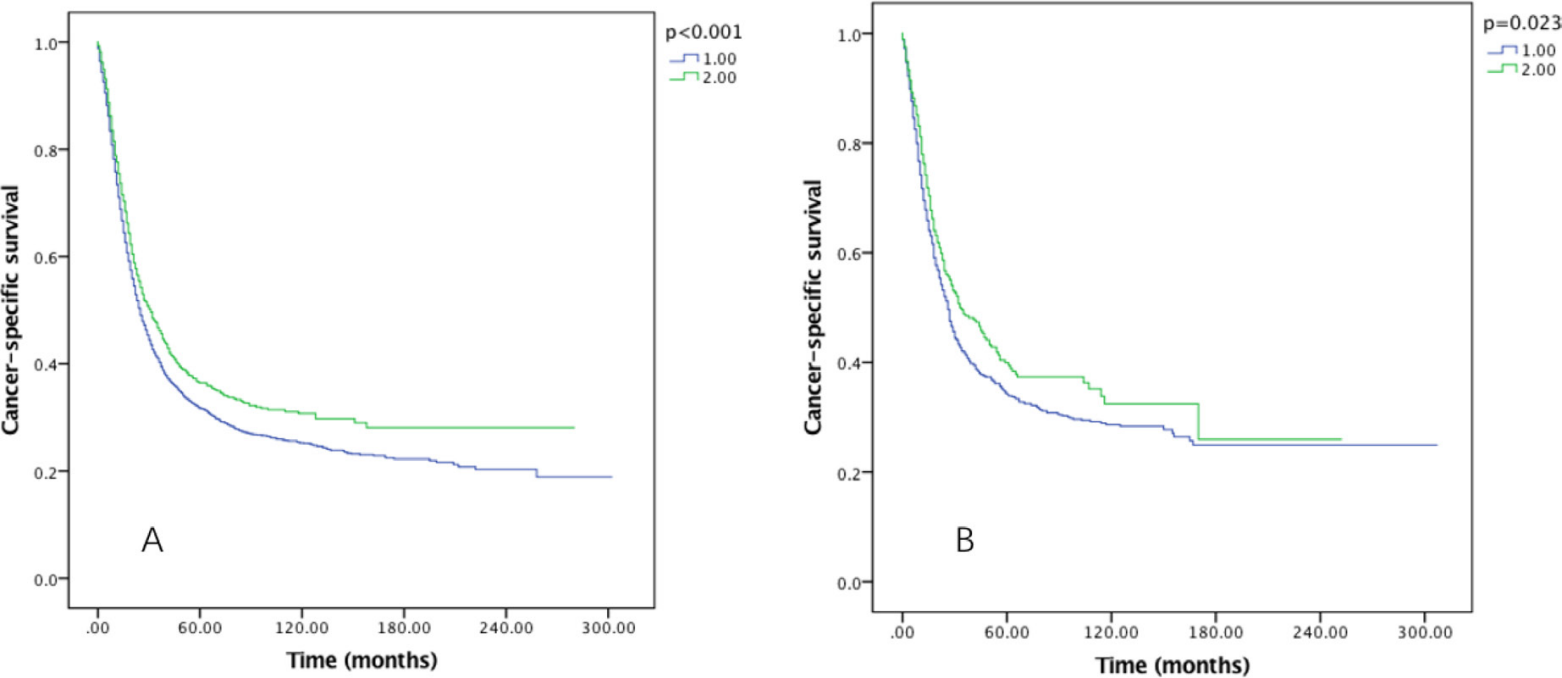
Cause-specific survival of Siewert type II GEJ cancer patients in different RLNs groups stratified by sex (**A**) male, (**B**) female. Kaplan-Meier curves illustrate the cause-specific survival of patients between 1–20 RLNs group and 21–90 RLNs group in male (A) and female (B). Significance was determined by log-rank analysis.

After examined the effect of RLNs on OS (Figure 3) and CSS (Figure 4) by sex, we found that the survival benefit was significantly better in Group 2 than in Group 1 both in male and female (*P* < 0.05).

**Figure 5:**
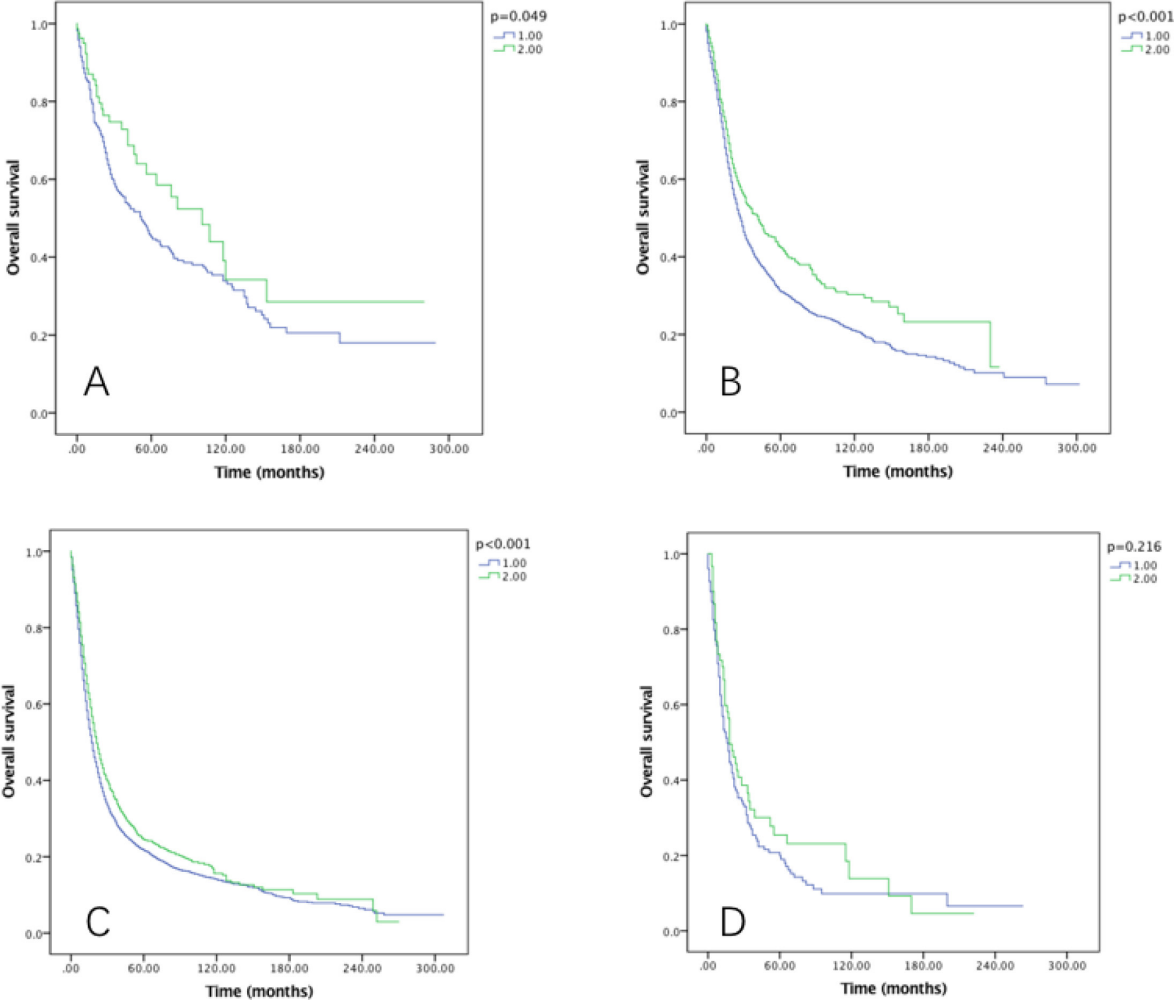
Overall survival of Siewert type II GEJ cancer patients in different RLNs groups stratified by grade (**A**) well differentiated, (**B**) moderately differentiated, (**C**) poorly differentiated, (**D**) undifferentiated. Kaplan-Meier curves illustrate the overall survival of patients between 1–20 RLNs group and 21–90 RLNs group in well differentiated (A), moderately differentiated (B), poorly differentiated and undifferentiated tumors. Significance was determined by log-rank analysis.

**Figure 6:**
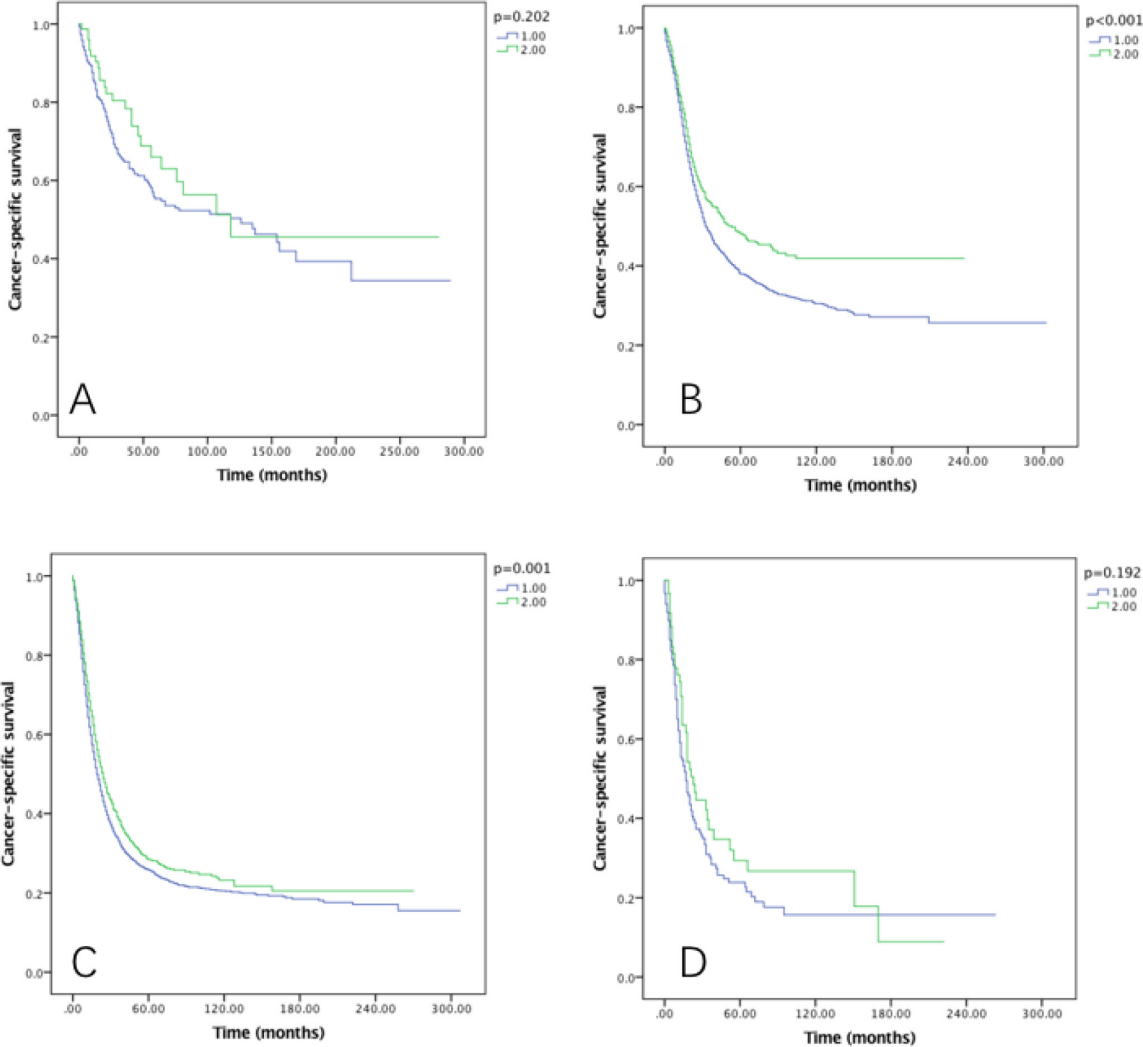
Cause-specific survival of Siewert type II GEJ cancer patients in different RLNs groups stratified by grade (**A**) well differentiated, (**B**) moderately differentiated, (**C**) poorly differentiated, (**D**) undifferentiated. Kaplan-Meier curves illustrate the cause-specific survival of patients between 1–20 RLNs group and 21–90 RLNs group in well differentiated (A), moderately differentiated (B), poorly differentiated and undifferentiated tumors. Significance was determined by log-rank analysis.

The prognostic effect of RLNs on OS and CSS by grade was also examined, and it was significantly associated with OS in well, moderately and poorly differentiated grade tumors (*P* < 0.005), but not associated with OS in undifferentiated grade tumor (Figure 5). As to the CSS, in moderately and poorly differentiated grade tumors, significant survival can be benefited from 21–90 RLNs Group (*P* < 0.05) (Figure 6).

**Figure 7:**
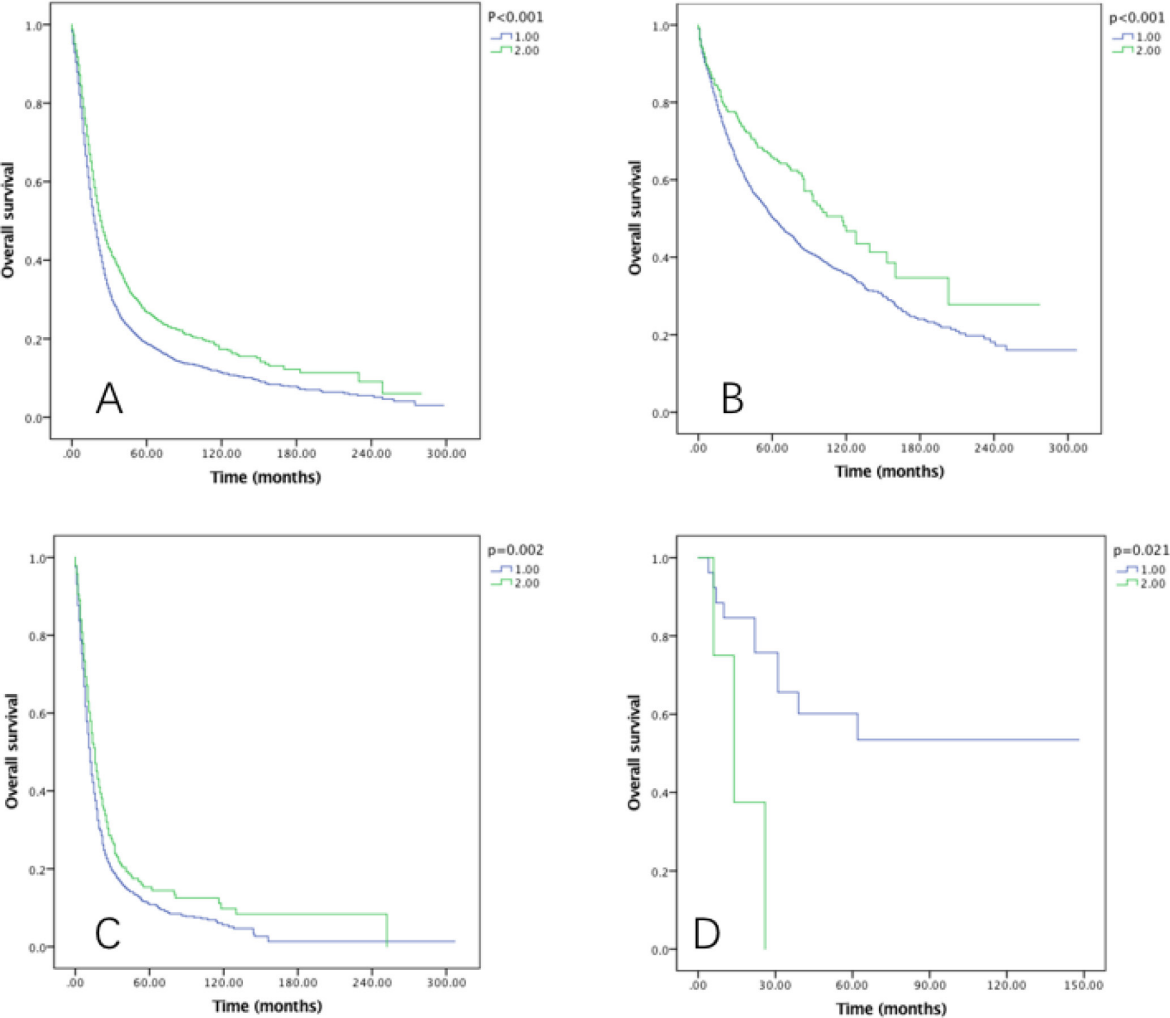
Overall survival of Siewert type II GEJ cancer patients in different RLNs groups stratified by SEER histology (**A**) reginal, (**B**) localized, (**C**) distant, (**D**) unstaged. Kaplan-Meier curves illustrate the overall survival of patients between 1–20 RLNs group and 21–90 RLNs group in reginal (A), localized (B), distant and unstaged tumors defined by SEER histology. Significance was determined by log-rank analysis.

**Figure 8:**
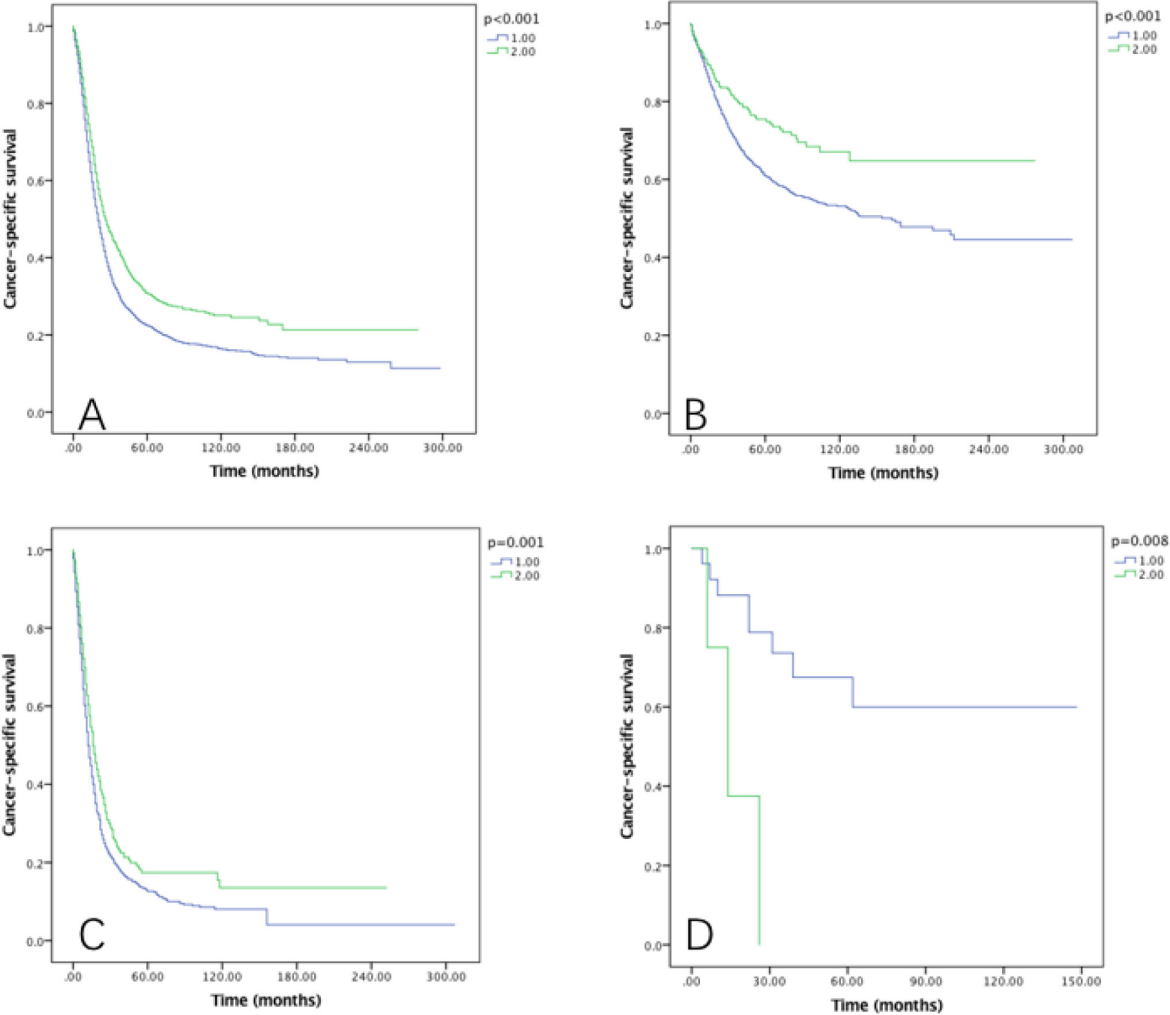
Cause-specific survival of Siewert type II GEJ cancer patients in different RLNs groups stratified by SEER histology (**A**) reginal, (**B**) localized, (**C**) distant, (**D**) unstaged. Kaplan-Meier curves illustrate the cause-specific survival of patients between 1–20 RLNs group and 21–90 RLNs group in reginal (A), localized (B), distant and unstaged tumors defined by SEER histology. Significance was determined by log-rank analysis.

Besides sex and grade, seer histology and tumor histology were also examined on their effect of RLNs on OS and CSS. Seer histology stage was divided into 4 subtypes (reginal, localized, distant and unstaged), and we found that among all these four subtypes, RLNs were significantly associated with OS (Figure 7) and CSS (Figure 8) (*P* < 0.05).

**Figure 9:**
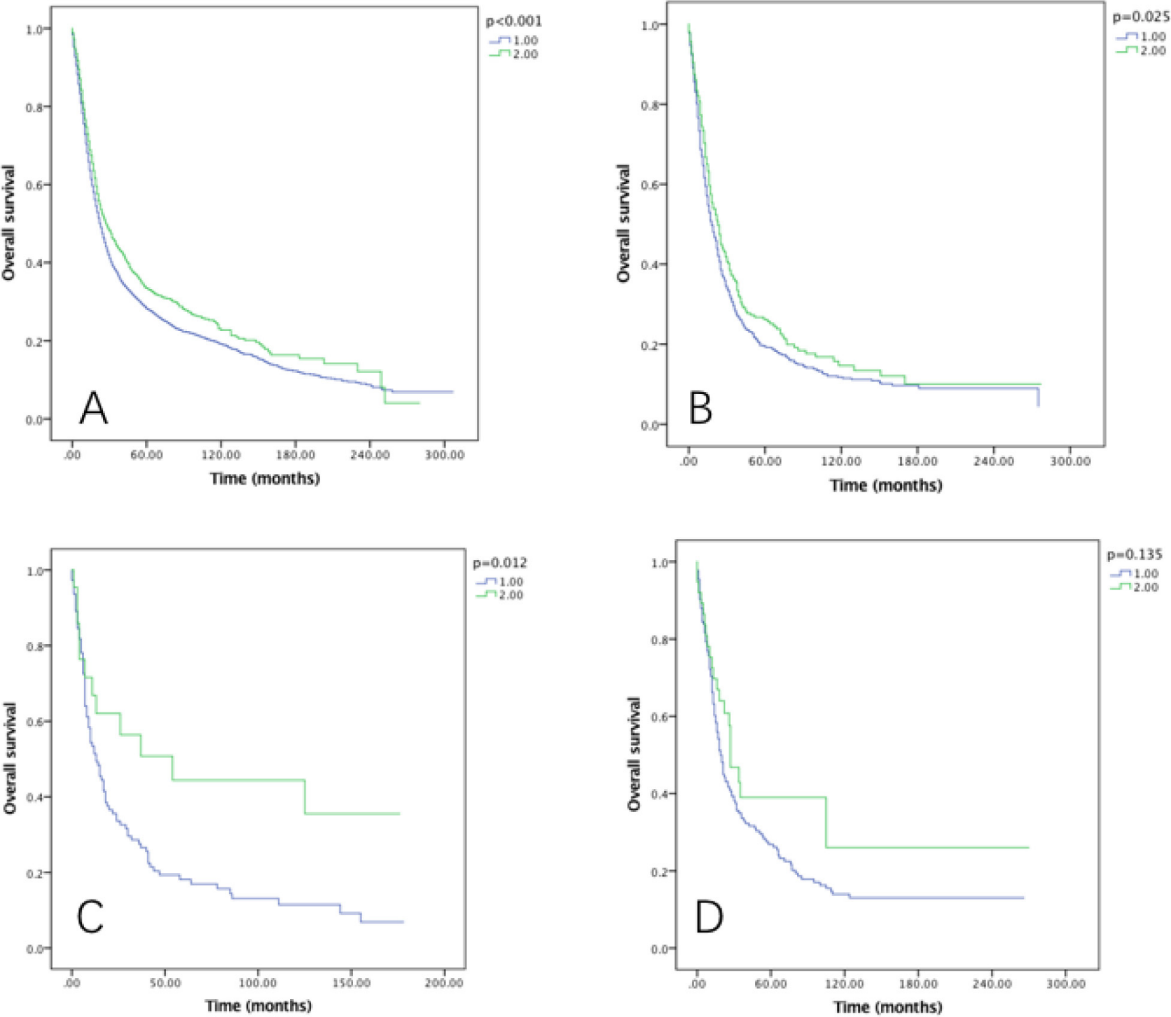
Overall survival of Siewert type II GEJ cancer patients in different RLNs groups stratified by tumor histology (**A**) adenocarcinoma, (**B**) cystic, (**C**) mucinous, (**D**) squamous. Kaplan-Meier curves illustrate the overall survival of patients between 1–20 RLNs group and 21–90 RLNs group in adenocarcinoma (A), cystic (B), mucinous and squamous carcinoma. Significance was determined by log-rank analysis.

**Figure 10:**
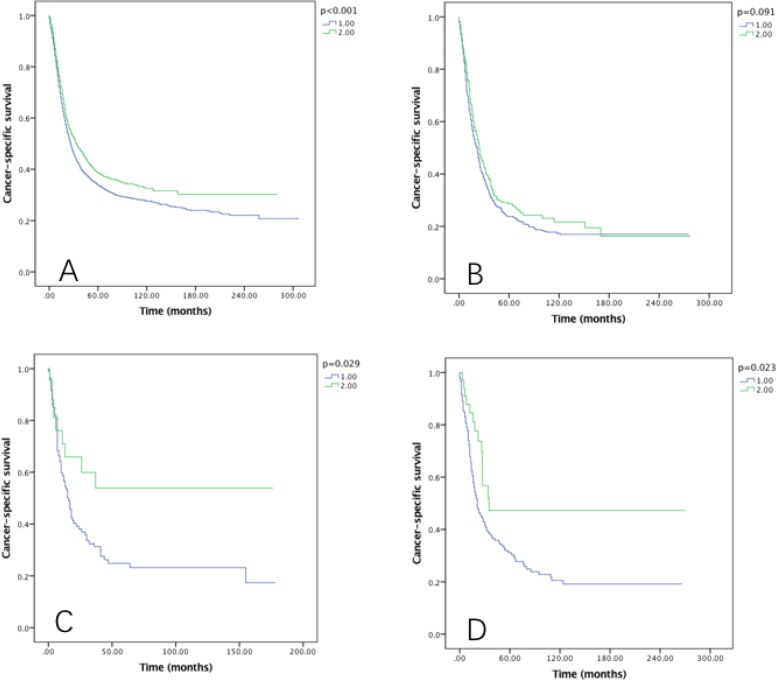
Cause-specific survival of Siewert type II GEJ cancer patients in different RLNs groups stratified by tumor histology (**A**) adenocarcinoma, (**B**) cystic, (**C**) mucinous, (**D**) squamous. Kaplan-Meier curves illustrate the cause-specific survival of patients between 1–20 RLNs group and 21–90 RLNs group in adenocarcinoma (A), cystic (B), mucinous and squamous carcinoma. Significance was determined by log-rank analysis.

As to the tumor histology, overall survival benefits can be achieved from Group 2 patients in adenocarcinoma, cystic, mucinous and squamous carcinoma (*P* < 0.05) (Figure 9), while CSS were associated with RLNs in adenocarcinoma, cystic and mucinous carcinoma (*P* < 0.05) (Figure 10).

## DISCUSSION

In recent years, an increasing trend of Siewert type II GEJ cancer was observed in the western country [20, 21]. Due to the aggressive behavior of GEJ cancers, it often resulted in a low survival rate. Currently the main treatment for GEJ cancers is surgery. However, surgical approaches were quite different among three Siewert type GEJ cancers. For Siewert type I and type II cancers, the main treatment mirrored the approaches for esophageal and gastric cancer, respectively. However, the standard procedure for Siewert type II cancer remains controversial [22].

Based on previous studies, the survival of Siewert type II cancers decreased significantly (53% to 11%) when there were lymph nodes (LNs) metastasis, which indicated LNs as a prognostic factor for this cancer [23]. Accordingly, adequate lymph nodes dissection may improve the prognosis of patients. The AJCC staging system suggested at least 15 lymph nodes examined for adequate staging in gastric cancer [19]. For oesophageal cancer the minimum number for resected LNs was not well defined [24], studies have reported the adequate number of LNs ranging from 10 to 40 [25–28]. As to the GEJ cancers, no large cohort study has been reported to validate the optimal RLNs number in Siewert type II cancer. Using the SEER database, this study is currently the largest study exploring the association between survival and the number of RLNs during surgery in Siewert type II GEJ cancer patients.

In this study, we demonstrated that the number of RLNs was an independent prognostic factor for overall survival and cancer-specific survival in Siewert type II GEJ cancer patients. 21 or more resected lymph nodes indicated better survival in Siewert type II GEJ cancer patients. Studies revealed that missing positive lymph nodes will lead to false negative results with poor survival rates [29]. Our study also revealed that with more resected lymph nodes (Group2), the LNR was significantly higher than inadequately resected lymph nodes (Group1) (*P* = 0.002), which further clarified that adequately removed LNs can result in accurate stage classification with proper intervention. In addition, we also observed age, year of diagnosis, sex, marital status, grade, seer histology stage, tumor histology, radiation and LNR were prognostic factors for Siewert type II cancer. After stratified by sex, grade, SEER histology and tumor histology, the number of RLNs is still consistently associated with OS and CSS. According to our results, we recommended surgeons should at least dissect 21 lymph nodes to achieve satisfying prognosis of Siewert type II patients.

Besides lymph node resection number, lymph node resection extension is also of great significance for stage and survival in GEJ cancers. Generally, esophagogastrectomy with two-field lymphadenectomy (abdominal and thoracic) was the standard surgical procedure for Siewert type I cancers, while in Siewert type III cancers formal D2 nodal dissection along with total gastrectomy was recommended [22]. The optimal extent of lymphadenectomy for Siewert type II cancers still remains controversial.

In Siewert type II cancers, the main affected lymph nodes were the paracardial and lesser curvature nodes, followed by nodes in the lower mediastinum, supra-pancreatic and greater curvature [30]. Stipa *et al.* suggested total gastrectomy with radical lymphadenectomy could lead to longer survival time [31], while other studies argued that limited lymphadenectomy (paracardial and less curvature nodes) with proximal gastrectomy enjoyed significant improved overall and cause-specific survival [30, 32, 33], which might be an alternative to radical surgical procedure.

In the study conducted by Omloo *et al.* [34], though patients with 1 to 8 positive lymph nodes benefit significantly from meaningful radical lymphadenectomy (41% increase in 5-year cause-specific survival), radical lymphadenectomy did not offer the same benefit in patients without positive lymph nodes or those with more than 8 positive lymph nodes.

Multimodality treatment including preoperative chemotherapy or radiation were validated to yield more satisfying survival outcomes than surgery alone in several studies in Siewert type II cancers. Van *et al*. enrolled 366 patients with esophageal and gastroesophageal junction cancers and revealed that preoperative chemoradiotherapy enjoyed significant improved 5-year survival than surgery alone (47% vs 24%) [6]. A phase III trial of trimodality therapy also found that the median survival time of GEJ cancer patients can be extended from a median of 1.79 years to 4.48 years, along with the 5-year survival rate elevated from 16% to 39.13% when chemotherapy and radiation was prescribed prior to surgery [35].

There were some limitations in this study. Firstly, the Seer database lacks the data on pathological stage, chemotherapy, the regimens of chemotherapy and other potentially influencing factors that might affect the survival. However, the Seer program offers the data of large number of patients, which may decrease the selection bias generated during single center analysis. Meanwhile, a large prospective multicenter study still needs to be implemented to validate the optimal numbers of RLNs in patients with Siewert type II cancer.

In conclusion, RLNs was an independent prognostic factor for Siewert type II GEJ cancer patients and patients can achieve better overall and cancer-specific survival with 21 or more LNs dissected.

## METHODS

### Patients

Data was retrieved from the SEER registry, which covered approximately 28 percent of the population of the United States. Patients with Siewert type II GEJ cancers from 1988 to 2013 were identified from the SEER registry. The SEER did not include the Siewert classification in the database, so we included patients satisfying the conditions of both CS Scheme entry of “EsophagusGEJunction” and Primary Site entry of “Cardia, NOS”. Patients without surgery or with multiple primary malignancies were excluded, those with no or unknown reginal lymph nodes examined were also excluded from the study. Informed consent was not required for the extraction of data from SEER program. This study was approved by the ethics committee of the Sir Run Run Shaw Hospital.

### Clinicopathological factors

The following clinicopathological factors were collected from the SEER database: age, sex, year of diagnosis, race, histological type, grade, seer historic stage, marital status at diagnosis, the number of RLNs, and the lymph node ratio (LNR). The number of RLNs was the total number of regional lymph nodes removed. The LNR was the ratio of the number of positive lymph node to the total number of RLNs. Vital status, cause of death and radiation were also recorded.

### Statistical analysis

The χ^2^ and Fisher's exact tests were used to analyze differences between qualitative data. Multivariate Cox regression analyses were generated to analyze risk factors for overall survival (OS) and cancer-specific survival (CSS). Survival rates were plotted using the Kaplan–Meier method and compared using the log-rank test. Lymph node ratio (LNR) between different resected lymph nodes (RLNs) groups were compared using Mann-Whitney *U* test for unpaired data. All data were analyzed with the SPSS statistical software package, version 23.0 (IBM Corporation, Armonk, NY, USA). A *p* value of < 0.05 was considered significant.

## Abbreviations

RLNs: Resected lymph nodes
GEJ: Gastroesophageal junction
SEER: Surveillance Epidemiology and End Results
OS: Overall survival
CSS: cause-specific survival
LNR: Lymph Node Ratio
LNs: lymph nodes
